# Advances in Editing Silkworms (*Bombyx mori*) Genome by Using the CRISPR-Cas System

**DOI:** 10.3390/insects13010028

**Published:** 2021-12-27

**Authors:** Gabriela-Maria Baci, Alexandra-Antonia Cucu, Alexandru-Ioan Giurgiu, Adriana-Sebastiana Muscă, Lilla Bagameri, Adela Ramona Moise, Otilia Bobiș, Attila Cristian Rațiu, Daniel Severus Dezmirean

**Affiliations:** 1 Faculty of Animal Science and Biotechnology, University of Animal Sciences and Veterinary Medicine Cluj-Napoca, 400372 Cluj-Napoca, Romania; gabriela-maria.baci@usamvcluj.ro (G.-M.B.); antonia.cucu@usamvcluj.ro (A.-A.C.); alexandru.giurgiu@usamvcluj.ro (A.-I.G.); adriana-sebastiana.musca@usamvcluj.ro (A.-S.M.); lilla.bagameri@usamvcluj.ro (L.B.); obobis@usamvcluj.ro (O.B.); ddezmirean@usamvcluj.ro (D.S.D.); 2 Faculty of Biology, University of Bucharest, 050095 Bucharest, Romania

**Keywords:** *Bombyx mori*, CRISPR-Cas, silkworms, genome engineering, insect biotechnology, entomology

## Abstract

**Simple Summary:**

One of the most powerful gene editing approaches is the CRISPR (clustered regularly interspaced short palindromic repeats)-Cas (CRISPR-associated) tool. The silkworm (*Bombyx mori*) has a great impact on the global economy, playing a pivotal role in the sericulture industry. However, *B. mori* came into the spotlight by representing one of science’s greatest contributors, being used to establish extraordinary bioreactors for the production of target proteins and illustrating a great experimental model organism. Herein, we focus on progress made in the field of *B. mori*’s genome manipulation by using CRISPR-Cas. In order to edit *B. mori*’s genome, remarkable advances were made, such as exposing gene functions and developing mutant lines that exhibit enhanced resistance against *B. mori* nucleopolyhedrovirus (BmNPV). We also discuss how CRISPR-Cas accelerated the fundamental investigation in *B. mori*, and beyond, thus highlighting the great potential of the insect’s biotechnology in numerous scientific fields.

**Abstract:**

CRISPR (clustered regularly interspaced short palindromic repeats)-Cas (CRISPR-associated) represents a powerful genome editing technology that revolutionized in a short period of time numerous natural sciences branches. Therefore, extraordinary progress was made in various fields, such as entomology or biotechnology. *Bombyx mori* is one of the most important insects, not only for the sericulture industry, but for numerous scientific areas. The silkworms play a key role as a model organism, but also as a bioreactor for the recombinant protein production. Nowadays, the CRISPR-Cas genome editing system is frequently used in order to perform gene analyses, to increase the resistance against certain pathogens or as an imaging tool in *B. mori*. Here, we provide an overview of various studies that made use of CRISPR-Cas for *B. mori* genome editing, with a focus on emphasizing the high applicability of this system in entomology and biological sciences.

## 1. Introduction

The life sciences research fields were revolutionized by the outstanding development of various genome editing tools. By using specific techniques of genome editing, the genomic DNA of every living organism can be submitted to guided changes, such as deletions, insertions, and sequence substitutions [1].

In recent years, several genome editing tools have been in the spotlight. Among them, there are three remarkable technologies, namely those relying on programmable nucleases (i.e., the transcription activator like effector nucleases (TALENs)), zinc finger nucleases (ZFNs), and clustered regularly interspaced short palindromic repeat - associated nucleases (CRISPR-Cas) [2,3]. Currently, by using engineered nucleases, remarkable advances are being made regarding the correction of genetic mutations, gene expression regulation, and the development of therapeutic agents; these approaches are also used for a better understanding of gene functions and the mechanisms underlying the development of certain genetic disorders or various diseases [4].

When it comes to genome editing, it is crucial to avoid off-target effects, but overall, the CRISPR-Cas system exhibits reliable results, owing to a great degree of fidelity [4,5]. Since its discovery in bacteria, the CRISPR-Cas system has been continuously exploited, representing an extremely versatile tool for the scientific community due to its reprogrammable feature. Currently, this system is used to edit the genomes of various organisms, such as bacteria, insects, plants, or human cells [6].

For more than 5000 years, humans have been involved in the domestication of one of the most economically important insects, namely *Bombyx mori*. Historically, *B. mori* gained prominence due to silk production, but now it is one of the most valued model organisms for life science branches. Just to mention a few recent achievements, *B. mori* has been used as a bioreactor, for recombinant proteins production [7,8,9,10], while its silk is used to produce extraordinary silk-based biomaterials that exhibit great importance for the medical field [11]. The silk proteome contains two major proteins, namely fibroin and sericin. The silk gland consists of three anatomical areas, namely the posterior (PSG), the middle (MSG), and the anterior (ASG) silk gland. Fibroin is synthesized in the PSG and sericin is assembled in the MSG [12].

In 2018, Ma et al. [13] excellently summarized the advances of using genome editing tools in silkworms. However, herein, we mainly focus on the potential of CRISPR-Cas technology in editing *B. mori*’s genome [13]. We navigate through the recent progresses in using the outstanding CRISPR-Cas system in *B. mori* and discuss the latest studies that utilized this approach in order to investigate the genes function, to regulate the gene expression and to enhance the resistance against *B. mori* nucleopolyhedrovirus (BmNPV). Additionally, the key role of *B. mori* in the scientific fields will be discussed.

## 2. The CRISPR-Cas System

CRISPR-Cas is one of the key methods employed by many molecular biology scientific laboratories. Since its first description [14], genome editing focused research was implemented by countless research groups [15,16,17].

### 2.1. The CRISPR-Cas Complex Role in the Immunity System

When investigating the *iap* gene product in the opportunistic pathogen *Escherichia coli*, Ishino et al. (1987) [18] observed an atypical structure, specifically the repetition of several homologous sequences. Later, this type of structure was observed in various bacterial, as well as archaeal strains [19,20]. Subsequently, these repetitive sequences were linked with exogenous genetic material, and following several years, their assembling mechanism and function were elucidated [21]. This type of sequence can be placed on the chromosomal DNA, but it can also be found on the plasmid DNA [22].

The scientists demonstrated that CRISPR-Cas, which is present in one-third of bacteria and nearly in all archaea, has a key role in host’s adaptive immunity. It protects the organism against various intruders, such as viruses, but it also offers protection against other mobile genetic elements, such as transposons or plasmids [23].

The CRISPR-Cas system structure includes three main components, i.e., the CRISPR arrays, the associated Cas proteins, and the leader nucleotide sequence. The first genetic component, the CRISPR locus, is characterized by identical repeats structures (21–37 bp) that are highly conserved and the spacer sequences that are acquired fragments of invader’s nucleic acid material. The CRISPR array is located downstream from *cas* genes. The latter encodes for Cas proteins that are crucial to the immune reaction [24].

Initially, only four distinct Cas proteins (1–4) were reported, due to the rapid evolution of biological sciences; currently, there numerous Cas proteins have been described [25,26], Cas1 being the most analyzed [27].

CRISPR-Cas has a great adaptability, with host-related specificities; thus, it exhibits a significant diversity. The varying feature is defined by the CRISPR array and the *cas* gene sequences. The classification of these types of systems is based on the signature Cas proteins. Currently, there are two major classes of CRISPR-Cas systems, each also di-vided in several groups [28]. Regarding the leader nucleotide sequence, it has been shown that it has a key role by carrying the essential promoter sequences for the transcription of CRISPR loci. Besides the promoter, the leader contains specific signals that are crucial for the adaptation stage from the first phase of CRISPR-Cas activation [29].

The adaptation is the first functional stage of the CRISPR-Cas mechanism, during which the foreign nucleic acid is recognized by several Cas proteins [30] and consequently integrated next to a leader sequence. Through this mechanism, in evolution, the spacers are arranged chronologically, and this feature helps bacteria and archaea to enhance their protection against the genetic material of the latest foreign encounter [31]. Each new acquired spacer is accompanied by a repeat sequence; therefore, the CRISPR array expands with every invasion [32].

The CRISPR array is transcribed in the second step, specifically in the biogenesis phase [33]. First of all, it is being transcribed into a precursor CRISPR RNA (crRNA). At the end of this phase, there are numerous mature crRNAs molecules, resulting from the action of RNase III that process the precursor crRNA. Each crRNA includes a spacer and a repeat sequence [31,34,35].

The last step of the system’s mechanism is the interference phase. It involves the degradation of the foreign nucleic acid, by targeting and cleaving it [36]. The products of the biogenesis phase, the crRNAs, act like guides for targeting the invader, which is then cleaved following a Cas proteins cascade that act like molecular scissors [37].

### 2.2. The CRISPR-Cas System as a Genome Editing Tool

When it comes to leading tools in genetic engineering, the CRISPR-Cas system can be considered the foremost instrument. After elucidating its function in various organisms, scientists aimed to exploit its versatility, in order to overcome the disadvantages of other available genome editing tools [38]. Even if scientific studies still report the use of ZFNs and TALENs techniques as editing tools, the CRISPR-Cas system is the most effective genome editing instrument, standing on top in regards to efficiency, cost-effectiveness, and the relative simplicity of use [39] (Table 1). Another considerable advantage of this system is represented by its capacity to simultaneously target multiple genes [40].

Of the numerous CRISPR-Cas systems, CRISPR-Cas9 is currently the most used instrument in laboratories across the world [47]. The Cas9 nuclease is the signature protein of CRISPR-Cas II systems and it is responsible for double strand DNA breaks [27]. Three different methods to deliver the Cas9 endonuclease have been described. It can be directly delivered by microinjection into the embryos, while the other two delivery methods involve a plasmid that expresses the Cas9 enzyme, or a messenger RNA (mRNA) sequence that encodes it. Of the three techniques, in terms of genome engineering, the earliest mentioned is the best option due to its certain advantages. By directly delivering the protein, low immunogen effects were observed. Furthermore, the off-target activity is minimized compared with the other two methods [48]. The use of CRISPR-Cas9 is a simple but powerful genome editing tool, with various implementations, and their impact on new research trends has been reviewed elsewhere [49].

The CRISPR-Cas9 mechanism relies on the Cas9 nuclease and a guide sequence (gRNA). As the name implies, the gRNA has the role to guide the Cas9 nuclease to a target site in order to cleave the DNA. The key feature of gRNA is the extensive complementarity with the target sequence [50].The protospacer-adjacent motif (PAM) bordering the target complementary sequence has a key role, since in its absence, the CRISPR-Cas systems would degrade their own CRISPR loci. In order to perform a cleavage, the Cas9 protein scans for the PAM sequence. Even if the gRNA is complementary with the target sequence, the Cas9 endonuclease will not cleave it in the absence of PAM [51].

The central factor that influences the success of the gene editing process is the repair path of the double-strand breaks produced by Cas9. There are two main repair pathways: the homology-directed repair (HDR) and the nonhomologous end joining (NHEJ), respectively [52]. More often, NHEJ is exploited in order to acquire indels mutations, specially to obtain small deletions. These deletions are extremely useful for disclosing gene functions [53]. However, the HDR machinery is used not just to obtain knock-out or knock-down mutations, such as the expected output following NHEJ, but to generate target knock-ins. Therefore, by using HDR, exogenous sequences can be successfully integrated into the host’s genome. Currently, major efforts are being made in order to enhance the sequence replacement by using the HDR mechanism [54].

**Figure 1:**
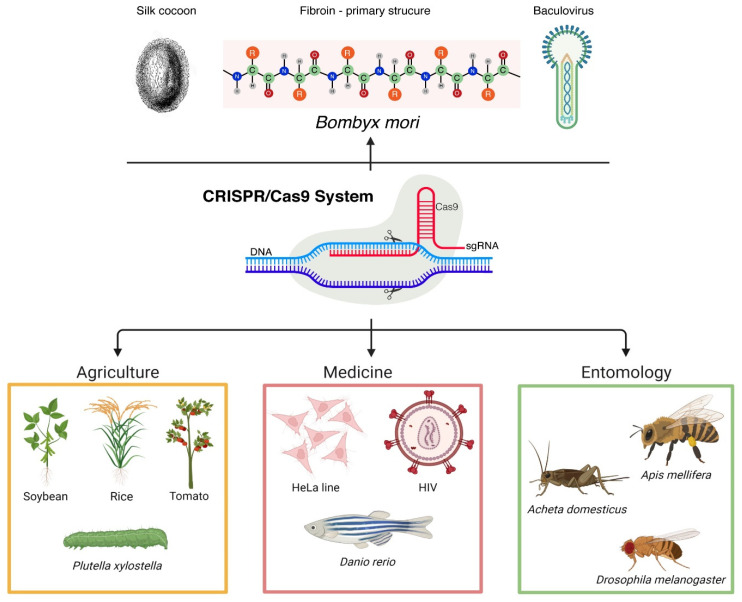
Schematic representation of the most important current applications of CRISPR-Cas9 in entomology, medicine, and agriculture. On top, a simplified description of CRISPR-Cas9 applicability in *B. mori* that is extensively described in the main text (created with BioRender.com, accessed on 2 December 2021).

CRISPR-Cas9 is currently used in multiple research fields, such as agriculture (editing of various agricultural plant genomes or pest insect’s genome) [55,56,57], biotechnology, food industry, and medicine (modeling diseases using HeLa cells, deciphering HIV infection mechanisms, using various experimental models, such as *Danio rerio* to tackle cancer and neurological diseases, etc.) [58,59], just to mention a few (Figure 1) [60].

### 2.3. CRISPR-Cas9 in Entomology

Being the most diverse and numerous category of organisms for decades [61], insects have been intensively studied. Countless studies have been performed due to insects’ key roles in ecology, agriculture, and medicine [62,63]. Considering this, numerous research groups aimed to use the CRISPR-Cas9 system to manipulate the insects’ genome. The first application of CRISPR-Cas9 was performed in *Drosophila melanogaster* [64] due to its strategic importance as arguably the main experimental model organism for life sciences [65]. Besides *D. melanogaster*, the researchers also used the CRISPR-Cas9 applicability on *B. mori*, *Apis mellifera*, *Aedes aegypti*, and *Tribolium castaneum* [66,67].

Gratz et al. (2013) [68] programmed CRISPR-Cas9 to edit *Drosophila’s* genome. The authors targeted the *yellow* gene, which is commonly used in various studies. First, they aimed to determine if this genome engineering tool could be efficient and could fulfill its role to induce breaks in the target sequence. By using the CRISPR-Cas9 system in *Drosophila*, not only was the *yellow* gene successfully knocked out, but the genome’s alterations were also germline transmitted. Subsequent to the deletion of the target gene, a donor sequence was designed. This sequence provided the template for the HDR repair pathway and its use was to test the accuracy of specific replacement of *yellow* gene with an exogenous sequence. These sequence replacements were transmitted to descendants as well. Their data showed that there was no off-target activity and it highlighted the feasibility of using the CRISPR-Cas9 technology in eukaryotes [68].

Aiming to further highlight the feasibility of choosing this system to perform genome alteration in *Drosophila*, Yu et al. (2013) [69] designed two gRNAs to induce mutations in two regions of the *yellow* gene. In addition, they targeted other six sequences, both euchromatic and heterochromatic loci. Remarkably, a definite mutation in *ms(3)k81* was transmitted to descendants in a proportion of 100%. By successfully targeting heterochromatic loci, their result showed that the CRISPR-Cas9 system is efficient for altering the heterochromatin [69]. *Drosophila* have been used in numerous studies in order to examine the insecticide resistance [70,71,72]. In this direction, Douris et al. (2020) [56] notably summarized the progress in using CRISPR-Cas9 to explore the genetic basis of this mechanism.

The CRISPR-Cas9 technique was used to perform functional analysis concomitantly on two genes belonging to the cricket (*Gryllus bimaculatus)* [73]. *G. bimaculatus* is an important insect for experimental studies; for example, it plays an important role for evolutionary developmental studies and comparative biology, but it is also a relevant model organism for neurobiology and behavioral sciences [74]. The efficiency of inserting a donor sequence via a homology-independent technique was tested in two *hox* genes, namely *Gb-Ubx* and *Gb-abd-A*. After inserting the donor fragment into essential exons of both genes, their function was lost. Thus, functional investigations of *hox* genes could be carried out by using the knock-in/knock-out approaches [73].

Being one of the most important social insects [75] and as it plays a crucial role as a pollinator, the honeybee (*Apis mellifera*) has been intensively studied. It also plays a pivotal role in various therapeutic areas due to honey production. This natural product has extraordinary benefits for human health, exhibiting antioxidant, antiviral, and antibacterial effects [76]. Due to its special characteristics, the use of honey is not limited to humans, but this natural product is being used to improve certain features of other insects, such as silkworms [77]. There are numerous studies that detail functional analysis of *A. mellifera* genes by exploiting the CRISPR-Cas9 system. For instance, Hu et al. (2019) [67] reported the successful utilization of this system for knocking out the *mrjp 1* gene from the honeybee genome. The CRISPR-Cas9 complex was delivered through microinjection and they tested two specific regions of embryos, for identifying the most convenient structure for delivering the gRNA and the Cas9 endonuclease. By microinjecting the construct at the dorsal posterior side, there was a low rate of successful manipulation (11.8%); however, when choosing the ventral cephalic side, the results showed a great rate of gene editing (93.3%). Trying to validate the previous results, the authors also targeted *pax6*. Based on the previously obtained results, they microinjected the CRISPR-Cas9 construct at the ventral cephalic side. The results showed an editing rate of 100% [67]. Targeting the same gene, *mrjp 1*, similar results have been obtained in another study [78]. Thus, functional analysis of *A. mellifera* genes can be effectively performed by using the CRISPR-Cas9 system.

Considering the same topic of gene function research, Nie et al. (2021) [79] used the CRISPR-Cas9 technology to determine if the *yellow-y* gene plays a crucial role in the process of cuticular melanin synthesis in *A. mellifera*. They targeted this gene due to its great potential for mutants screening, being a selectable marker. By disrupting it, the phenotype of worker cuticle has changed, mainly due to the black pigment decreasing, thus confirming the *yellow-y* gene critical role in melanin pigmentation. However, as future prospects, this could be a great genetic marker for upcoming genomic research [79].

Referring to *A. mellifera* sex determination, it is controlled by the heterozygosity at a particular locus that harbors the key *complementary sex determiner* (*csd*) gene. The bees that are heterozygous at this specific locus are females, while the males are homozygous or hemizygous [80]. In a recent study, Wang et al. (2021) [81] used the CRISPR-Cas9 tool in order to knock out the *csd* gene and, thus, eliminated the genetic difference between females and males. Subsequently, they aimed to observe the transcriptome difference between the two sexes in this particular genetic background. They also successfully induced target mutations in mutant haploid individuals. It was observed that the expression level of several male-biased genes was higher in the mutant males. On the other hand, the expression level of several specific female-biased genes was lower. Their data also confirmed that *csd* interacts with certain genes, such as *fruitless*, *troponin T*, and *transformer-2* just to mention a few [81].

## 3. *Bombyx mori*

For numerous reasons, *B. mori* is one of the most studied insects, especially because it presents a real interest for the scientific community. It has been completely domesticated and plays the pivotal role in sericulture, being reared principally for silk production on a large scale [82]. Considering the great role of silk in the textile industry and its use as a biomaterial in medicine, major efforts are being made to enhance silk’s quality, but also to increase its quantity [12]. Moreover, there are numerous studies that describe the process of obtaining enhanced silk with appreciable properties by genetically manipulating the silkworms [83,84,85,86]. *B. mori*’s genome was manipulated in order to enhance silk’s properties. For instance, by knocking in the major ampullate silk protein from spiders into *B. mori*’s genome, a research group obtained silk with superior mechanical characteristics [84].

*B. mori* is an oligophagous insect and its main food source are the mulberry leaves, a nutritional preference that influences its biological and economical parameters. There is a major drawback when it comes to this source of nutrition, namely the limited availability of mulberry leaves. In order to be able to rear silkworms not only in spring and summer, artificial diets are currently being used for their nourishment [77,87].

Regardless of human medicine advances, certain microorganisms developed survival strategies and numerous infectious diseases remain a crucial problem worldwide [88]. In this context, *B. mori* is a reliable experimental model and exhibits the great advantage of a short development cycle that consists of four different stages. The first period of growth is the egg phase, followed by the larvae, pupa, and moth phases. The larvae phase plays a crucial role in silkworm’s development, being the longest phase and including five different stages. Another significant advantage of using *B. mori* is the fact that in the last instar larvae, the body size has nearly 5 cm; hence, it is facile to manipulate or to exploit it for various purposes. Moreover, this size also facilitates the body’s dissection; thus, multiple target tissues or organs can easily be obtained [89,90].

### B. mori as a Model Organism

The level of attention received as a model experimental organism increased, since several groups made available *B. mori*’s genome data [91]. Various mutant strains have been described and the genetic analysis confirmed the numerous genetic traits. Another important aspect is that its manipulation is not associated with ethical concerns [90].

Being susceptible to various infectious agents, *B. mori* is one of the most used experimental models for drug screening, evaluation of different virulence factors, and identifying the pathogen genes responsible for its virulence [90]. Hitherto, several studies used *B. mori* to evaluate the effectiveness of antibiotics against certain human pathogens [92,93,94]. In a recent study, silkworms were used to examine the efficacy of three different glycopeptide antibiotics against *Staphylococcus aureus* infection. As a result, authors highlighted the great feasibility and efficacy of using *B. mori* to mimic bacterial infections in order to examine the therapeutic potential of antibiotics [95]. Moreover, recently, silkworms were used to evaluate the impact on several antibacterial compounds against *Cutibacterium acnes* [96]. As an experimental model organism, *B. mori* is currently being intensively used for various other purposes, as detailed in Table 2.

## 4. Applications of CRISPR-Cas in *B. mori*

The first communication of successful manipulation of *B. mori*’s genome by using the CRISPR-Cas9 tool, was reported by Wang et al. (2013) [113]. The authors targeted an essential gene [113], *BmBlos2*, that is orthologous to the *Blos2* human gene [114]. Two sgRNAs (23-bp) were designed to induce mutations leading to loss of target gene function. Each complex formed by one sgRNA and the Cas9 nuclease was injected in the preblastoderm embryonic stage. Ordinarily, the larval integument is opaque, but when the *BmBlos2* gene function is lost, the tegument becomes translucent. This effect could be viewed as a phenotypic marker for mutant’s detection. Of all individuals, 94% and 95.6%, respectively were successfully edited by using the two sgRNAs. This study highlighted the feasibility of using CRISPR-Cas9 not only in *B. mori*, but also in other lepidopteran insects. These findings are of great interest by revealing CRISPR-Cas9 system’s applicability in pest control approaches [113].

The multiplexable potential of CRISPR-Cas9 technology was highlighted by Liu et al. (2014) [115]. First, the *BmBlos2* gene was targeted for site specific mutagenesis, in order to confirm the feasibility of using the CRISPR-Cas9 system in *B. mori*. Following this, other six genes were targeted to confirm the system’s multiplexable feature: *tyrosine hydroxylase*, *red egg*, *yellow-e*, *kynureninase*, *ebony*, and *flugellos*. Mutations were induced in each target gene, without evidence for the system’s off-target activity [115]. The multiplexable feature of CRISPR-Cas9 is facilitating the process of genome engineering by simultaneously and precisely inducing mutations in different sites. This property of CRISPR-Cas9 enables researchers to perform precise and elaborate target mutagenesis in a time-effective manner [116].

Other approaches aimed to knock out the *BmKu70* gene by using CRISPR-Cas9 in order to target its second exon [117]. The *BmKu70* gene is coding a highly conserved protein, Ku70, which plays a key role in numerous mechanisms: cell adhesion, apoptosis, the maintenance of telomeres length, etc. In addition, numerous studies reported that by inactivating the *BmKu70* gene, the frequency of homologous repair is increased. In order to test this hypothesis, the authors performed a transient analysis in genetically manipulated embryos. They knocked in the *Bm702* gene that is found on the Z chromosome. Their results confirmed that by knocking out *BmKu70* the homologous repair frequency is expanded. These promising results are of great interest for fundamental research in *B. mori*. [117].

It has been demonstrated by Fujinaga et al. (2017) [118] that in *B. mori*, the insulin-like growth factor-like peptide (IGFLP), is closely related to the genital disc, particularly involved in its growth. Due to the lack of studies performed in vivo on this topic, the same research group aimed to confirm the role of IGFLP by inactivating it [119]. For this purpose, they used the CRISPR-Cas9 genome editing tool. The absence of IGFLP leads to smaller ovaries and a lower number of laid eggs compared with the wild type. On the other hand, the size of laid eggs and its development were not affected. These findings indicated that this hormone has no impact on *B. mori*’s fertility. The ecdysteroids play a crucial role in IGFLPs production, by inducing gene expression. Furthermore, it has been shown previously that ecdysteroids have a key role in ovary development. Therefore, the authors initially appraised that a low ecdysteroids titre caused reduced ovary weights. However, by analyzing the transgenic females, their data showed that the ecdysteroids titre was the same. This study reveals insights on IGFLPs impact on ovarian development [119].

In a recent study [120], a research group explored the effect of activating the *BmFibH* gene in the *B. mori* embryonic cells. For this purpose, they constructed a complex that involved the inactive form of Cas9 nuclease (dCas9) and a VPR activating domain, driven by a certain promoter. This activating domain consists of several different activators: VP64, p65, and Rta, respectively. When it comes to sgRNAs, three specific constructs were designed to target the promoter of the gene of interest. In order to confirm the success of *BmFibH* activation, first they determined its expression in normal embryonic cells. Their results showed that the *BmFibH* gene is strongly downregulated in untransformed cells. On the other hand, the transfected cells exhibited a higher *BmFibH* expression level. Moreover, their data showed that the activation of the target gene impacted the cellular stress responses [120]. Cui et al. (2018) [121] targeted the *BmFibH* gene in order to explore its role in the development of the silk gland. After the CRISPR-Cas9 construct was designed, a total number of 630 eggs were microinjected, but only 12.5% hatched. After analyzing the unhatched eggs, they observed that all embryos were genetically edited. By knocking out the *BmFibH*, severe changes were observed, such as naked pupa or thin cocoons. All individuals that exhibited naked pupae died. Moreover, by inactivating this gene, several other genes involved in the processes of degradation, such as the autophagy, were upregulated. These findings offer a better understanding of FibH protein’s role in silk gland development [121].

Keeping in mind the feasibility of expressing spider silk genes in *B. mori* [84], in order to obtain enhanced silk, Zhang et al. (2019) [122] used CRISPR-Cas9 to acquire high-performance fibers. By using this technique, the authors successfully knocked in spider silk genes in silkworms’ genome. Accordingly, they designed two types of systems, FibL-CRISPR-Cas9 and FibH-CRISPR-Cas9. To avoid disrupting the protein production, spider silk genes were knocked in into one of the introns of *BmFibL* or *BmFibH*. They demonstrated the feasibility of employing CRISPR-Cas9 *in B. mori* to obtain silk with enhanced mechanical properties at industrial scale. The strategy described in this study can be further used to obtain numerous exogenous proteins that exhibit great interest for medical applications and beyond [122].

The microRNAs (miRNAs) are key regulators when it comes to gene expression. Notably, they recognize by complementarity specific mRNAs and inactivate them [123]. It has been revealed that *miR-2* is one of the most important miRNAs for wing morphogenesis in *D. melanogaster* and exhibits great influence on the Notch pathway. The *BmAwd* and *BmFng* genes are known positive regulators of this signaling pathway and are also potential *miR-2* targets. On this topic, Ling et al. (2015) [124] used the CRISPR-Cas9 technology to investigate the function of *miR-2* in *B. mori*. In the first phase of the study, the authors used the Gal4/UAS system to overexpress the *miR-2*, resulting in deformed adults’ wings. However, in the second phase of the research, the CRISPR-Cas9 system was used to knock out the two *miR-2* target genes. The loss of function of *BmFng* and *BmAwd* also led to deformed wings. Both phases of the study confirmed that in silkworms, the *miR-2* plays a crucial role in wing development [124].

In another study, Liu et al. (2020) [125] used the CRISPR-Cas9 system to explore the function of *miR-34*, another miRNA that exhibits a great impact on insect development. Firstly, they overexpressed *miR-34* in a transgenic line constructed by using a pBac plasmid. The *miR-34* overexpression negatively impacted the body size and wing morphology. Secondly, the CRISPR-Cas9 system was used to inactivate the *miR-34* by using two different gRNAs. The *miR-34* ablation led to larvae developmental delay. Using several bioinformatic tools, they predicted that the *miR-34* target genes could be *BmE74*, *BmCpg4*, *BmLcp*, *BmWcp11,* and *BmBrc-z2*. Various analyses revealed that miR-34 target genes are just *BmE74* and *BmCpg4.* While it is well known that *BmE74* plays a key role in growth and morphogenesis, functional analysis of the second gene had to be performed. Given this, the CRISPR-Cas9 system was used to knock out *BmCpg4* resulting in affected wings, thus highlighting the gene’s role in wing development [125].

Regarding the silkworms’ innate defense mechanisms, there are two major activation pathways involved in the expression of numerous evolutionarily conserved antimicrobial peptides (AMPs) genes, and the Toll and the Imd pathways [126,127]. When Gram-negative bacterial or fungal contamination occurs, the Toll pathway is activated. One of the key genes that is involved in the Toll pathway is *BmCactus*. Being a negative regulator, once the infection with the mentioned pathogens occurs, this gene is being phosphorylated and inactivated. Considering this, Park et al. (2018) [128] used the CRISPR-Cas9 technology to perform site-target mutagenesis targeting the *BmCactus* gene in a specific *B. mori* cell line. The authors designed six different gRNAs and transfected the CRISPR-Cas9 complex in the *B. mori* ovarian cell line by electroporation, but observed a very low survival rate of only 24%. Their data showed that all gRNAs determined site induced mutagenesis. By disrupting the *BmCactus* gene, the expression of several antimicrobial proteins (e.g., lysozyme and lebocin) was stimulated [128]. Keeping in mind the great importance of AMPs in clinical research [129], this study underlined the outstanding potential of *B. mori* usage in life science fields and the feasibility of using the advanced CRISPR-Cas9 genetic scissors to edit genomes for different purposes [128].

Ecdysteroids are steroid hormones that play a crucial role in the process of molting and metamorphosis in insects. The most important molting hormone is 20-hydroxyecdysone (20E) [130], but even if its biosynthesis processes have been intensively studied, the 20E metabolism has not been well-documented. However, there are several genes that are believed to be involved in the inactivation of 20E, but their biological functions have not been fully understood. Therefore, Li et al. (2015) [131] used *B. mori* to investigate the biological functions of one particular 20E inactivation enzyme, specifically the ecdysone oxidase (EO). Having a great impact on insects’ key processes, it is crucial to regulate the ecdysteroids concentration. Accordingly, the EO participates in ecdysteroids’ oxidation [132]. The CRISPR-Cas9 system was used to deplete the *BmEo* gene, and consequently, there was observed that the duration of the fifth instar larval phase was prolonged by 24 h [131].

Another key element that is involved in insects’ development is the juvenile hormone (JH) [133]. The central role in the JH degradation process is played by the juvenile hormone esterase (JHE). Zhang et al. (2017) [134] used CRISPR-Cas9 in *B. mori* to deplete the encoding gene for JHE, specifically *BmJhe*, in order to investigate its function. Their data showed that by knocking out this gene, the fourth and fifth instar stages were prolonged due the fact that JH metabolism was delayed. These findings are not only important for functional analysis, but also for the sericulture industry. For silk production, it is of great impact to expand the larval stages, because this leads to larger larvae, and thus, larger cocoons are produced. These findings highlight the feasibility of using genome editing tools for economic purposes [134].

Moreover, CRISPR-Cas was used in *B. mori* to perform epigenetic modifications. In this context, Liu et al. (2019) [135] explored the impact of methylation on silkworms’ development. This study is of great significance by providing a strategy for the investigation of DNA methylation importance in a target locus. Furthermore, their study represents a starting point for exploring the impact of DNA methylation status on different phenotypes in silkworms and beyond [135]. Furthermore, Xing et al. (2019) [136] used this technology for labeling endogenous regions in *B. mori* embryonic cell line and targeted the *BmFibH* gene. Using CRISPR-dCas9 as an imaging tool has extraordinary impact on performing fundamental research, but also on providing insights on insecticide resistance [136].

As for the detection of CRISPR-Cas induced mutations, there are a plethora of screening methods. On this topic, in a recent study, Brady et al. (2020) [137] described a new approach and provided a protocol for screening, characterizing, and stabilizing the mutant silkworm lines. The provided protocol involves several molecular methods that allow the recognition of the induced mutations on both autosomes and sex chromosomes [137].

In Table 3, several studies are described that used the CRISPR-Cas technology in order to genetically edit *B. mori*.

### Applicability of CRISPR-Cas in Anti-BmNPV Therapy

Viruses represent a major threat to numerous hosts, including humans or insects, being one of the most rapidly mutating biological entities. However, there is an urgent need to develop new methods to combat these pathogens. Being one of the most valued molecular tools, the CRISPR-Cas system is currently being used for developing antiviral strategies in various organisms [155,156,157]. Of all nucleases described in the specialized literature, it has been shown that different variants of Cas9, Cas12, and Cas13 exhibit the most promising results regarding the antiviral approaches [155].

In respect to insect virology, BmNPV was the first discovered pathogen (1998) [158]. In the sericulture industry, this baculovirus causes extensive economic losses; therefore, it has been intensively studied. Being part of *Baculoviridae* family, its infection leads to the most severe disease and only a few silkworm strains are resistant to this virus [159]. Currently, there are several traditional methods that help to enhance silkworms’ resistance to BmNPV, but they exhibit serious limitations [158,160,161].

However, CRISPR-Cas has been successfully used as an antiviral therapy in *B. mori*, especially against BmNPV. Chen et al. (2017) [162] selected two genes that are involved in baculovirus’ replication and propagation processes: *immediate early-1 (ie-1*) and *me53,* respectively. The authors designed two gRNA for each target gene. The engineered plasmid contained three expression cassettes. One cassette contained the Cas9 nuclease, the second one included the gRNAs and the last one harbored a selecting marker, specifically the enhanced green fluorescent protein (EGFP). Even if the silkworms’ viability and fecundity was not impacted, the transgenic homozygotes experienced a delay of the larval stage development. After performing viral inoculation in both wild type group and transgenic group, it was observed that the second category posed great viral resistance against BmNPV [162]. Likewise, another group of researchers targeted two other genes, *ie-0* and *ie-2*, by using CRISPR-Cas9. After being inoculated with occlusion bodies of BmNPV, the survival rate reached 65% for the transgenic silkworms [163].

The multiplexable feature of the CRISPR-Cas9 technology was underlined in a study performed by Dong et al. (2019) [164]. The researchers targeted in BmNPV three different genes essential for viral replication: *ie-1*, the *major envelope glycoprotein* and the *late expression factor-11*. The success of this study revealed one promising strategy in inhibiting different *B. mori* by using the powerful CRISPR-Cas method.

## 5. Conclusions

*B. mori* is one of the most important domesticated insects due to its great potential as a biotechnological platform to produce recombinant proteins, but also because of its great success as an experimental model organism [92,110]. Due to its extraordinary prospects, a wide range of studies have been performed that accelerated fundamental research and beyond in silkworms. Being the most feasible technology in terms of genome editing, the CRISPR-Cas system is currently used in many laboratories specialized in medicine, agriculture, alimentary industry, and entomology research [34,165]. A hallmark of CRISPR-Cas is the relative ease of designing CRISPR-based experiments. As reviewed elsewhere, there are numerous bioinformatics tools that facilitate the guide RNA design, as well as the prediction and evaluation of editing results [166]. In our experience, a thorough guide RNA design can also be achieved by using standard sequence alignment tools and manual inspection of potential target regions. Considering the aforementioned, the utilization of the CRISPR-Cas system as a gene editing tool augmented the research in *B. mori*. Most of the studies have been focused on using the CRISPR-Cas system to perform functional gene analysis, to elucidate certain mechanisms [135,148], or to enhance silkworms’ resistance to BmNPV [164]. By reviewing the most remarkable work in this field, we provide deep insights that offer support for future research not only in *B. mori*, but also in other insect experimental models.

There are interesting future prospects when it comes to using the CRISPR-Cas technology in silkworms. Nowadays, by genetically manipulating the *B. mori* genome, major progress is being made for a better understanding of the process of fibroin and sericin synthesis, but also great advances are made to increase the understanding in the most important processes in silkworms. Notable great applications of CRISPR-Cas in *B. mori* refer to the development of enhanced silk fibers and the production of recombinant proteins that exhibit importance for various scientific fields. Even if compared with the other genome editing techniques, CRISPR-Cas9 exhibits lower off-target activity, although currently, it could not be confirmed that these unfavorable effects are completely eliminated. However, there is a major need to eliminate or at least reduce the off-target activity.

## Figures and Tables

**Table 1:** Comparison between TALEN, ZFN, and CRISPR-Cas gene editing technologies.

Traits	TALEN	ZFN	CRISPR-Cas	References
Origin	Prokaryotic	Eukaryotic	Prokaryotic	[41]
Efficiency (%)	76	12	81	[42]
Specificity	Moderate	Low	High	[43,44,45,46]
Target site recognition	Any site	Any site	Pam motif (NGG) required	[43]
Multiplex potential	Low	Low	High	[43,45]
Processing time	Time consuming	Time consuming	Short	[45]
Methylation sensitive	Sensitive	Sensitive	Not sensitive	[42]
Engineering feasibility	Moderate/High	Moderate	Moderate/High	[42,45]
Dimerization required	Yes	Yes	No	[44]
Cost effectiveness	Moderate	No	Yes	[43,44]

**Table 2:** Summary of various uses of *B. mori* as an experimental model organism.

Type of Model Organism	Brief Description	Purpose	References
Human disease model	Transgenic *B. mori* expressing *hIR* (human insulin receptor)	Drug evaluation for diabetes treatment	[97,98]
	Bacterial (*Listeria monocytogenes*) infection model in *B. mori*	Evaluating the interaction between host and pathogen; investigating the activity of vitamin A against microbial infections	[99]
	Fungal (*C. albicans*) infection model in silkworms	Assessing the *C. albicans* biofilm development	[100]
	Inducing deletions in the *BmSpr* gene, leading to sepiapterin reductase deficiency	Treatment options evaluations against sepiapterin reductase deficiency	[101]
Model for pesticide toxicity	Exposing silkworms to phoxim	Identifying specific biomarkers for phoxim stress; evaluating the toxicity reaction and the pretreatment with nanoparticulate titanium dioxide	[102]
	Inducing genotoxicity by feeding the silkworms with different doses of avermectin	Exploration of certain genes that are required for the DNA repairing mechanism	[103]
	Treating *B. mori* larvae with Fenvalerate-20EC	Evaluation of Fenvalerate-20EC impact on several digestive enzymes	[104]
Model for drugs toxicity	Injecting the silkworms with three different pharmacologically active agents (4-methyl umbelliferone, 7-ethoxycoumarine)	Evaluation of the metabolic pathway of these compounds	[92]
	Exposing the silkworms to fungal infections	Exploring pharmacokinetic parameters of an antifungal agent, Voriconazole	[105]
	Injecting cytotoxic drugs into *B. mori* larvae	Evaluation of cytotoxic drugs impact	[106]
Model for nanomaterials toxicity	Spreading silver nanoparticles on mulberry leaves	Toxicity evaluation of silver nanoparticles	[107,108,109,110]
	Injecting subcutaneously zinc oxide nanoparticles	Evaluation of zinc oxide nanoparticles toxicity, accumulation, and distribution	[111]
	Injecting in the dorsal vein different nanoparticles with great interest in various life science branches	Investigation of different silicon and carbon nanomaterials toxicity level against hemocytes	[112]

**Table 3:** CRISPR-Cas applications in *B. mori*.

Target Gene	Mutation Type	Delivery Approach	Objective	Gene Function	References
*BmTim*	Deletions	Plasmid	Functional gene analysis	Exhibit an impact on the embryo hatching process	[138]
*BmApp*	Deletions, insertions	mRNA	Functional gene analysis	Regulates wing development and cell mitosis	[139]
*BmFoxo*	Deletions	Plasmid	Analysis of *BmFoxo* and JH interaction	Involved in JH degradation	[140]
*BmKmo*	Deletions	Protein	Phenotypic analysis	Involved in the process of egg formation and eye coloring	[141]
*BmYki*	Deletions	Plasmid	Functional gene analysis	Involved in organ development and regeneration	[142]
*BmTorso*	Deletions	Plasmid	Functional gene analysis	Maintain the steroid hormones balance	[143]
*BmEsp*	Deletions	Plasmid	Functional gene analysis	Involved in a female’s reproducibility	[144]
*BmTudor*	Deletions, insertions	Plasmid	Investigating the frequency of homologous recombination	Included in the stress granule formation	[145]
*BmIdgf*	Deletions	mRNA	Analyzing the pigmentation mechanism	Plays a key role in the melanization mechanism	[146]
*BmBngr-a2*	Deletions	Plasmid	Exploring functional studies of certain ion transport peptides	Involved in water homeostasis	[147]
*BmTctp*	Deletions	Plasmid	Functional analysis	Involved in different cell process, such as growth, development, and proliferation	[148]
*BmGr66*	Deletions	Plasmid	A better understanding of the specific feeding preference	Involved in silkworms’ specific feeding preferences	[149]
*BmOvo*	Deletions	Plasmid	Functional analysis	Involved in germline sex determination and wing metamorphosis	[150]
*BmPhyhd1*	Deletions	Protein	Functional analysis	Exhibits a great impact on certain features of the epithelial cells	[151]
*BmWnt1*	Deletions	mRNA	Functional analysis	Involved in the embryogenesis	[152]
*BmE75b*	Deletions	mRNA	Functional analysis	Controls the developmental timing	[153]
*BmOrco*	Deletions	Plasmid	Exploration of adult mating behavior	Involved in silkworms’ olfactory system, being an odorant receptor co-receptor	[154]

## Data Availability

Not applicable.

## References

[1] Moon S.B., Kim D.Y., Ko J.H., Kim Y.S. (2019). Recent advances in the CRISPR genome editing tool set. Exp. Mol. Med..

[2] Guha T.K., Wai A., Hausner G. (2017). Programmable Genome Editing Tools and their Regulation for Efficient Genome Engineering. Comput. Struct. Biotechnol. J..

[3] Li H., Yang Y., Hong W., Huang M., Wu M., Zhao X. (2020). Applications of genome editing technology in the targeted therapy of human diseases: Mechanisms, advances and prospects. Signal Transduct. Target. Ther..

[4] Schuijff M., De Jong M.D.T., Dijkstra A.M. (2021). AQ methodology study on divergent perspectives on CRISPR-Cas9 in the Netherlands. BMC Med. Ethics.

[5] Zhang D., Hussain A., Manghwar H., Xie K., Xie S., Zhao S., Larkin R.M., Qing P., Jin S., Ding F. (2020). Genome editing with the CRISPR-Cas system: An art, ethics and global regulatory perspective. Plant Biotechnol. J..

[6] Li P., Wang L., Yang J., Di L., Li J. (2021). Applications of the CRISPR-Cas system for infectious disease diagnostics. Expert Rev. Mol. Diagn..

[7] Sezutsu H., Sumitani M., Kondo M., Kobayashi I., Takasu Y., Suzuki T., Yonemura N., Iizuka T., Uchino K., Tamura T. (2018). Construction of a Platform for the Development of Pharmaceutical and Medical Applications Using Transgenic Silkworms. Yakugaku Zasshi J. Pharm. Soc. Jpn..

[8] Kurihara H., Sezutsu H., Tamura T., Yamada K. (2007). Production of an active feline interferon in the cocoon of transgenic silkworms using the fibroin H-chain expression system. Biochem. Biophys. Res. Commun..

[9] Nakaya H., Tatematsu K.I., Sezutsu H., Kuwabara N., Koibuchi N., Takeda S. (2020). Secretory expression of thyroid hormone receptor using transgenic silkworms and its DNA binding activity. Protein Expr. Purif..

[10] Itoh K., Kobayashi I., Nishioka S., Hidaka T., Tsuji D., Sezutsu H. (2016). A transgenic silkworm overexpressing human lysosomal enzyme as a novel resource for producing recombinant glycobiologics and its application to development of enzyme replacement therapy for lysosomal diseases. Mol. Genet. Metab..

[11] Xu H., O’Brochta D.A. (2015). Advanced technologies for genetically manipulating the silkworm *Bombyx mori*, a model lepidopteran insect. Proc. R. Soc. B Biol. Sci..

[12] Montali A., Romanelli D., Cappellozza S., Grimaldi A., De Eguileor M., Tettamanti G. (2017). Timing of autophagy and apoptosis during posterior silk gland degeneration in *Bombyx mori*. Arthropod Struct. Dev..

[13] Ma S.Y., Smagghe G., Xia Q.Y. (2018). Genome editing in *Bombyx mori*: New opportunities for silkworm functional genomics and the sericulture industry. Insect Sci..

[14] Jinek M., Chylinski K., Fonfara I., Hauer M., Doudna J.A., Charpentier E. (2012). A Programmable Dual-RNA—Guided DNA endonuclease in adaptive bacterial immunity. Science.

[15] Nidhi S., Anand U., Oleksak P., Tripathi P., Lal J.A., Thomas G., Kuca K., Tripathi V. (2021). Novel CRISPR—Cas Systems: An Updated Review of the Current Achievements, Applications, and Future Research Perspectives. Int. J. Mol. Sci..

[16] Hahn F., Loures L.S., Sparks C.A., Kanyuka K., Nekrasov V. (2021). Efficient CRISPR/Cas-Mediated Targeted Mutagenesis in Spring and Winter Wheat Varieties. Plants.

[17] Hesami M., Yoosefzadeh Najafabadi M., Adamek K., Torkamaneh D., Jones A.M.P. (2021). Synergizing off-target predictions for in silico insights of CENH3 Knockout in Cannabis through CRISPR/Cas. Molecules.

[18] Ishino Y., Shinagawa H., Makino K., Amemura M., Nakata A. (1987). Nucleotide Sequence of the iap Gene, Responsible for Alkaline Phosphatase Isozyme Conversion in Escherichia coli, and Identification of the Gene Product. J. Bacteriol..

[19] Gophna U., Brodt A. (2012). CRISPR/Cas systems in archaea. Mob. Genet. Elem..

[20] Horvath P., Barrangou R. (2010). CRISPR/Cas, the immune system of Bacteria and Archaea. Science.

[21] Shabbir M.A.B., Shabbir M.Z., Wu Q., Mahmood S., Sajid A., Maan M.K., Ahmed S., Naveed U., Hao H., Yuan Z. (2019). CRISPR-cas system: Biological function in microbes and its use to treat antimicrobial resistant pathogens. Ann. Clin. Microbiol. Antimicrob..

[22] Rath D., Amlinger L., Rath A., Lundgren M. (2015). The CRISPR-Cas immune system: Biology, mechanisms and applications. Biochimie.

[23] Faure G., Shmakov S.A., Yan W.X., Cheng D.R., Scott D.A., Peters J.E., Makarova K.S., Koonin E.V. (2019). CRISPR–Cas in mobile genetic elements: Counter-defence and beyond. Nat. Rev. Microbiol..

[24] Mcdonald N.D., Regmi A., Morreale D.P., Borowski J.D., Boyd E.F. (2019). CRISPR-Cas systems are present predominantly on mobile genetic elements in Vibrio species. BMC Genom..

[25] Haft D.H., Selengut J., Mongodin E.F., Nelson K.E. (2005). A Guild of 45 CRISPR-Associated (Cas) Protein Families and Multiple CRISPR/Cas Subtypes Exist in Prokaryotic Genomes. PLoS Comput. Biol..

[26] Makarova K.S., Haft D.H., Barrangou R., Brouns S.J.J., Charpentier E., Horvath P., Moineau S., Mojica F.J.M., Wolf Y.I., Yakunin A.F. (2011). Evolution and classification of the CRISPR-Cas systems. Nat. Rev. Microbiol..

[27] Makarova K.S., Koonin E.V. (2015). Annotation and Classification of CRISPR-Cas Systems. CRISPR.

[28] Koonin E.V., Makarova K.S. (2019). Origins and evolution of CRISPR-Cas systems. Philos. Trans. R. Soc. B.

[29] Alkhnbashi O.S., Shah S.A., Garrett R.A., Saunders S.J., Costa F., Backofen R. (2016). Characterizing leader sequences of CRISPR loci. Bioinformatics.

[30] Alkhnbashi O.S., Costa F., Shah S.A., Garrett R.A., Saunders S.J., Backofen R. (2014). CRISPRstrand: Predicting repeat orientations to determine the crRNA-encoding strand at CRISPR loci. Bioinformatics.

[31] McGinn J., Marraffini L.A. (2018). Molecular mechanisms of CRISPR–Cas spacer acquisition. Nat. Rev. Microbiol..

[32] Sorek R., Lawrence C.M., Wiedenheft B. (2013). CRISPR-Mediated Adaptive Immune Systems in Bacteria and Archaea. Annu. Rev. Biochem..

[33] Roberts A., Barrangou R. (2020). Applications of CRISPR-Cas systems in lactic acid bacteria. FEMS Microbio. Rev..

[34] Hryhorowicz M., Lipiński D., Zeyland J., Słomski R. (2016). CRISPR/Cas9 Immune System as a Tool for Genome Engineering. Arch. Immunol. Ther. Exp..

[35] Terns M.P., Terns R.M. (2011). CRISPR-based adaptive immune systems. Curr. Opin. Microbiol..

[36] Newsom S., Parameshwaran H.P., Martin L., Rajan R. (2021). The CRISPR-Cas Mechanism for Adaptive Immunity and Alternate Bacterial Functions Fuels Diverse Biotechnologies. Front. Cell. Infect. Microbiol..

[37] Marraffini L.A., Sontheimer E.J. (2010). CRISPR interference: RNA-directed adaptive immunity in bacteria and archaea. Nat. Rev. Genet..

[38] Ishino Y., Krupovic M., Forterre P. (2018). History of CRISPR-Cas from Encounter with a Mysterious Repeated Sequence to Genome Editing Technology. J. Bacteriol..

[39] Sontheimer E.J., Barrangou R. (2015). The Bacterial Origins of the CRISPR Genome-Editing Revolution. Hum. Gene Ther..

[40] El-Mounadi K., Morales-Floriano M.L., Garcia-Ruiz H. (2020). Principles, Applications, and Biosafety of Plant Genome Editing Using CRISPR-Cas9. Front. Plant Sci..

[41] Agustın-Pavon C., Isalan M. (2014). Synthetic biology and therapeutic strategies for the degenerating brain. Bioessays.

[42] Chen L., Tang L., Xiang H., Jin L., Li Q., Dong Y., Wang W., Zhang G. (2014). Advances in genome editing technology and its promising application in evolutionary and ecological studies. Gigascience.

[43] Tavakoli K., Pour-Aboughadareh A., Kianersi F., Poczai P., Etminan A., Shooshtari L. (2021). Applications of CRISPR-Cas9 as an Advanced Genome Editing System in Life Sciences. BioTech.

[44] Guha T.K., Edgell D.R. (2017). Applications of Alternative Nucleases in the Age of CRISPR/Cas9. Int. J. Mol. Sci..

[45] Khan S.H. (2019). Genome-Editing Technologies: Concept, Pros, and Cons of Various Genome-Editing Techniques and Bioethical Concerns for Clinical Application. Mol. Ther. Nucleic Acids.

[46] Chira S., Gulei D., Hajitou A., Zimta A.A., Cordelier P., Berindan-Neagoe I. (2017). CRISPR/Cas9: Transcending the Reality of Genome Editing. Mol. Ther. Nucleic Acids.

[47] Manghwar H., Lindsey K., Zhang X., Jin S. (2019). CRISPR/Cas System: Recent Advances and Future Prospects for Genome Editing. Trends Plant Sci..

[48] Li J., Shi Y., Wu J., Li H., Smagghe G., Liu T. (2021). CRISPR/Cas9 in lepidopteran insects: Progress, application and prospects. J. Insect Physiol..

[49] Tyagi S., Kumar R., Das A., Won S.Y., Shukla P. (2020). CRISPR-Cas9 system: A genome-editing tool with endless possibilities. J. Biotechnol..

[50] Zhang Y., Showalter A.M. (2020). CRISPR/Cas9 Genome Editing Technology: A Valuable Tool for Understanding Plant Cell Wall Biosynthesis and Function. Front. Plant Sci..

[51] Collias D., Beisel C.L. (2021). CRISPR technologies and the search for the PAM-free nuclease. Nat. Commun..

[52] Yang H., Ren S., Yu S., Pan H., Li T., Ge S., Zhang J., Xia N. (2020). Methods Favoring Homology-Directed Repair Choice in Response to CRISPR/Cas9 Induced-Double Strand Breaks. Int. J. Mol. Sci..

[53] Bernheim A., Calvo-villamañán A., Basier C., Cui L., Rocha E., Touchon M., Bikard D. (2017). Inhibition of NHEJ repair by type II-A CRISPR-Cas systems in bacteria. Nat. Commun..

[54] Di Stazio M., Foschi N., Athanasakis E., Gasparini P., d’Adamo A.P. (2021). Systematic analysis of factors that improve homologous direct repair (HDR) efficiency in CRISPR/Cas9 technique. PLoS ONE.

[55] Zhu H., Li C., Gao C. (2020). Applications of CRISPR–Cas in agriculture and plant biotechnology. Nat. Rev. Mol. Cell Biol..

[56] Douris V., Denecke S., Van Leeuwen T., Bass C., Nauen R., Vontas J. (2020). Using CRISPR/Cas9 genome modification to understand the genetic basis of insecticide resistance: Drosophila and beyond. Pestic. Biochem. Physiol..

[57] Tyagi S., Kesiraju K., Saakre M., Rathinam M., Raman V., Pattanayak D., Sreevathsa R. (2020). Genome Editing for Resistance to Insect Pests: An Emerging Tool for Crop Improvement. ACS Omega.

[58] Yang Y., Xu J., Ge S., Lai L. (2021). CRISPR/Cas: Advances, Limitations, and Applications for Precision Cancer Research. Front. Med..

[59] Yang Y., Liu X., Li S., Chen Y., Zhao Y., Wei Y., Qiu Y., Liu Y., Zhou Z., Han J. (2020). Genome-scale CRISPR screening for potential targets of ginsenoside compound K. Cell Death Dis..

[60] Xu Y., Li Z. (2020). CRISPR-Cas systems: Overview, innovations and applications in human disease research and gene therapy. Comput. Struct. Biotechnol. J..

[61] Ma X., He K., Shi Z., Li M., Li F., Chen X.-X. (2021). Large-Scale Annotation and Evolution Analysis of MiRNA in Insects. Genome Biol. Evol..

[62] Brady D., Grapputo A., Romoli O., Sandrelli F. (2019). Insect Cecropins, Antimicrobial Peptides with Potential Therapeutic Applications. Int. J. Mol. Sci..

[63] Romoli O., Mukherjee S., Mohid S.A., Dutta A., Montali A., Franzolin E., Brady D., Zito F., Bergantino E., Rampazzo C. (2019). Enhanced Silkworm Cecropin B Antimicrobial Activity against Pseudomonas aeruginosa from Single Amino Acid Variation. ACS Infect. Dis..

[64] Cui Y., Sun J., Yu L. (2017). Application of the CRISPR gene-editing technique in insect functional genome studies—A review. Entomol. Exp. Appl..

[65] De Lazzari F., Sandrelli F., Whitworth A.J., Bisaglia M. (2020). Antioxidant Therapy in Parkinson’s Disease: Insights from Drosophila melanogaster. Antioxidants.

[66] Taning C.N.T., Van Eynde B., Yu N., Ma S., Smagghe G. (2017). CRISPR/Cas9 in insects: Applications, best practices and biosafety concerns. J. Insect Physiol..

[67] Hu X.F., Zhang B., Liao C.H., Zeng Z.J. (2019). High-Efficiency CRISPR/Cas9-Mediated Gene Editing in Honeybee (*Apis mellifera*) Embryos. G3 Genes Genomes Genet..

[68] Gratz S.J., Cummings A.M., Nguyen J.N., Hamm D.C., Donohue L.K., Harrison M.M., Wildonger J., O’Connor-giles K.M. (2013). Genome Engineering of Drosophila with the CRISPR RNA-guided Cas9 nuclease. Genetics.

[69] Yu Z., Ren M., Wang Z., Zhang B., Rong Y.S., Jiao R., Gao G. (2013). Highly Efficient Genome Modifications Mediated by CRISPR/Cas9 in Drosophila. Genetics.

[70] Perry T., Batterham P. (2018). Harnessing model organisms to study insecticide resistance. Curr. Opin. Insect Sci..

[71] Homem R.A., Davies T.G.E. (2018). An overview of functional genomic tools in deciphering insecticide resistance. Curr. Opin. Insect Sci..

[72] Scott J.G., Buchon N. (2019). Drosophila melanogaster as a powerful tool for studying insect toxicology. Pestic. Biochem. Physiol..

[73] Matsuoka Y., Nakamura T., Watanabe T., Barnett A.A., Noji S., Mito T., Extavour C.G. (2021). Establishment of CRISPR/Cas9-based knock-in in a hemimetabolous insect: Targeted gene tagging in the cricket *Gryllus bimaculatus*. bioRxiv.

[74] Donoughe S., Extavour C.G. (2016). Embryonic development of the cricket *Gryllus bimaculatus*. Dev. Biol..

[75] Evans J.D., Aronstein K., Chen Y.P., Hetru C., Imler J., Jiang H., Kanost M., Thompson G.J., Zou Z., Hultmark D. (2006). Immune pathways and defence mechanisms in honey bees *Apis mellifera*. Insect Mol. Biol..

[76] Cucu A.A., Baci G.M., Moise A.R., Dezsi S., Marc B.D., Stângaciu S., Dezmiream D.S. (2021). Towards a Better Understanding of Nutritional and Therapeutic Effects of Honey and Their Applications in Apitherapy. Appl. Sci..

[77] Baci G., Cucu A., Moise A.R., Dezmirean D.S. (2021). Applicability of Honey on Silkworms (*Bombyx mori*) and Quality Improvement of Its Biomaterials. Appl. Sci..

[78] Kohno H., Suenami S., Takeuchi H., Sasaki T., Kubo T. (2016). Production of Knockout Mutants by CRISPR/Cas9 in the European Honeybee, *Apis mellifera* L. Zool. Sci..

[79] Nie H., Liang L., Li Q., Li Z., Zhu Y., Guo Y., Zheng Q., Lin Y., Yang D., Li Z. (2021). CRISPR/Cas9 mediated knockout of Amyellow-y gene results in melanization defect of the cuticle in adult *Apis mellifera*. J. Insect Physiol..

[80] Gempe T., Hasselmann M., Schiøtt M., Hause G., Otte M., Beye M. (2009). Sex Determination in Honeybees: Two Separate Mechanisms Induce and Maintain the Female Pathway. PLoS Biol..

[81] Wang X., Lin Y., Liang L., Geng H., Zhang M., Nie H., Su S. (2021). Transcriptional Profiles of Diploid Mutant *Apis mellifera* Embryos after Knockout of csd by CRISPR/Cas9. Insects.

[82] Horie Y., Watanabe H. (1980). Recent advances in sericulture. Annu. Rev. Entomol..

[83] Saviane A., Romoli O., Bozzato A., Freddi G., Cappelletti C., Rosini E., Cappellozza S., Tettamanti G., Sandrelli F. (2018). Intrinsic antimicrobial properties of silk spun by genetically modified silkworm strains. Transgenic Res..

[84] Xu J., Dong Q., Yu Y., Niu B., Ji D., Li M., Huang Y., Chen X., Tan A. (2018). Mass spider silk production through targeted gene replacement in *Bombyx mori*. Proc. Natl. Acad. Sci. USA.

[85] Tang X., Ye X., Wang X., Zhao S., Wu M., Ruan J., Zhong B. (2021). High mechanical property silk produced by transgenic silkworms expressing the spidroins PySp1 and ASG1. Sci. Rep..

[86] Leem J.W., Fraser M.J., Kim Y.L. (2020). Transgenic and Diet-Enhanced Silk Production for Reinforced Biomaterials: A Metamaterial Perspective. Annu. Rev. Biomed. Eng..

[87] Cappellozza L., Cappellozza S., Saviane A., Sbrenna G. (2005). Artificial diet rearing system for the silkworm *Bombyx mori* (Lepidoptera: Bombycidae): Effect of vitamin C deprivation on larval growth and cocoon production. Appl. Entomol. Zool..

[88] Marrazzo P., Crupi A.N., Alviano F., Teodori L., Bonsi L. (2019). Exploring the roles of MSCs in infections: Focus on bacterial diseases. J. Mol. Med..

[89] Abdelli N., Peng L., Keping C. (2018). Silkworm, *Bombyx mori*, as an alternative model organism in toxicological research. Environ. Sci. Pollut. Res..

[90] Meng X., Zhu F., Chen K. (2017). Silkworm: A promising model organism in life science. J. Insect Sci..

[91] Yokoi K., Tsubota T., Jouraku A., Sezutsu H., Bono H. (2021). Reference Transcriptome Data in Silkworm *Bombyx mori*. Insects.

[92] Hamamoto H., Tonoike A., Narushima K., Horie R., Sekimizu K. (2009). Silkworm as a model animal to evaluate drug candidate toxicity and metabolism. Comp. Biochem. Physiol. Part C Toxicol. Pharmacol..

[93] Hamamoto H., Kurokawa K., Kaito C., Kamura K., Razanajatovo I.M., Kusuhara H., Santa T., Sekimizu K. (2004). Quantitative Evaluation of the Therapeutic Effects of Antibiotics Using Silkworms Infected with Human Pathogenic Microorganisms. Antimicrob. Agents Chemother..

[94] Kaito C., Sekimizu K. (2007). A silkworm model of pathogenic bacterial infection. Drug Discov. Ther..

[95] Montali A., Berini F., Brivio M.F., Mastore M., Saviane A., Cappellozza S., Marinelli F., Tettamanti G. (2020). A Silkworm Infection Model for In Vivo Study of Glycopeptide Antibiotics. Antibiotics.

[96] Matsumoto Y., Tateyama Y., Sugita T. (2021). Evaluation of Antibacterial Drugs Using Silkworms Infected by Cutibacterium acnes. Insects.

[97] Matsumoto Y., Ishii M., Ishii K., Miyaguchi W., Horie R., Inagaki Y., Hamamoto H., Tatematsu K.-I., Uchino K., Tamura T. (2014). Transgenic silkworms expressing human insulin receptors for evaluation of therapeutically active insulin receptor agonists. Biochem. Biophys. Res. Commun..

[98] Matsumoto Y., Ishii M., Hayashi Y., Miyazaki S., Sugita T., Sumiya E., Sekimizu K. (2015). Diabetic silkworms for evaluation of therapeutically effective drugs against type II diabetes. Sci. Rep..

[99] Castillo Y., Suzuki J., Watanabe K., Shimizu T., Watarai M. (2016). Effect of Vitamin A on Listeria monocytogenes Infection in a Silkworm Model. PLoS ONE.

[100] Matsumoto Y., Kurakado S., Sugita T. (2021). Evaluating Candida albicans biofilm formation in silkworms. Med. Mycol..

[101] Jiang G., Song J., Hu H., Tong X., Dai F. (2020). Evaluation of the silkworm lemon mutant as an invertebrate animal model for human sepiapterin reductase deficiency. R. Soc. Open Sci..

[102] Wang L., Su M., Zhao X., Hong J., Yu X., Xu B., Sheng L., Liu N., Shen W., Li B. (2015). Nanoparticulate TiO 2 Protection of Midgut Damage in the Silkworm (*Bombyx mori*) Following Phoxim Exposure. Arch. Environ. Contam. Toxicol..

[103] Shen W., Zhao X., Wang Q., Niu B., Liu Y., He L., Weng H., Meng Z., Chen Y. (2011). Genotoxicity evaluation of low doses of avermectin to hemocytes of silkworm (*Bombyx mori*) and response of gene expression to DNA damage. Pestic. Biochem. Physiol..

[104] Vyjayanthi N., Subramanyam M.V.V. (2002). Effect of Fenvalerate-20EC on Sericigenous Insects: II. Digestive Enzymes in the Nutritive Physiology of Silkworm, *Bombyx mori* L. Ecotoxicol. Environ. Saf..

[105] Yasu T., Matsumoto Y., Sugita T. (2021). Pharmacokinetics of voriconazole and its alteration by Candida albicans infection in silkworms. J. Antibiot..

[106] Inagaki Y., Matsumoto Y., Kataoka K., Matsuhashi N., Sekimizu K. (2012). Evaluation of drug-induced tissue injury by measuring alanine aminotransferase (ALT) activity in silkworm hemolymph. BMC Pharmacol. Toxicol..

[107] Pandiarajan J., Jeyarani V., Balaji S., Krishnan M. (2016). Silver Nanoparticles an Accumulative Hazard in Silkworm: *Bombyx mori*. Austin J. Biotechnol. Bioeng..

[108] Gad A.A. (2020). Toxicity effect of Silver Nanoparticles to the Haemocytes and Antioxidant activity of Silkworm *Bombyx mori*. Physiol. Entomol..

[109] Meng X., Abdlli N., Wang N., Lü P., Nie Z., Dong X., Lu S., Chen K. (2017). Effects of Ag Nanoparticles on Growth and Fat Body Proteins in Silkworms (*Bombyx mori*). Biol. Trace Elem. Res..

[110] Nouara A., Lü P., Chen L., Pan Y., Yang Y., Chen K. (2018). Silver effects on silkworm, *Bombyx mori*. J. Toxicol. Sci..

[111] Xu Y., Wang W., Ma L., Cui X., Lynch I., Wu G. (2020). Acute toxicity of Zinc Oxide nanoparticles to silkworm (*Bombyx mori* L.). Chemosphere.

[112] Li K.L., Zhang Y.H., Xing R., Zhou Y.F., Chen X.D., Wang H., Song B., Sima Y.H., Xu S.Q. (2017). Different toxicity of cadmium telluride, silicon, and carbon nanomaterials against hemocytes in silkworm, *Bombyx mori*. RSC Adv..

[113] Wang Y., Li Z., Xu J., Zeng B., Ling L., You L., Chen Y., Huang Y., Tan A. (2013). The CRISPR/Cas System mediates efficient genome engineering in *Bombyx mori*. Cell Res..

[114] Fujii T., Daimon T., Uchino K., Banno Y., Katsuma S., Sezutsu H., Tamura T., Shimada T. (2010). Transgenic analysis of the BmBLOS2 gene that governs the translucency of the larval integument of the silkworm, *Bombyx mori*. Insect Mol. Biol..

[115] Liu Y., Ma S., Wang X., Chang J., Gao J., Shi R., Zhang J., Lu W., Liu Y., Zhao P. (2014). Highly efficient multiplex targeted mutagenesis and genomic structure variation in *Bombyx mori* cells using CRISPR/Cas9. Insect Biochem. Mol. Biol..

[116] Sakuma T., Nishikawa A., Kume S., Chayama K., Yamamoto T. (2014). Multiplex genome engineering in human cells using all-in-one CRISPR/Cas9 vector system. Sci. Rep..

[117] Ma S., Chang J., Wang X., Liu Y., Zhang J., Lu W., Gao J., Shi R., Zhao P., Xia Q. (2014). CRISPR/Cas9 mediated multiplex genome editing and heritable mutagenesis of BmKu70 in *Bombyx mori*. Sci. Rep..

[118] Fujinaga D., Kohmura Y., Okamoto N., Kataoka H., Mizoguchi A. (2017). Insulin-like growth factor (IGF)-like peptide and 20-hydroxyecdysone regulate the growth and development of the male genital disk through different mechanisms in the silkmoth, *Bombyx mori*. Insect Biochem. Mol. Biol..

[119] Fujinaga D., Shiomi K., Yagi Y., Kataoka H., Mizoguchi A. (2019). An insulin-like growth factor-like peptide promotes ovarian development in the silkmoth *Bombyx mori*. Sci. Rep..

[120] Hu W., Wang X., Ma S., Peng Z., Cao Y., Xia Q. (2021). CRISPR-Mediated Endogenous Activation of Fibroin Heavy Chain Gene Triggers Cellular Stress Responses in *Bombyx mori* Embryonic Cells. Insects.

[121] Cui Y., Zhu Y., Lin Y., Chen L., Feng Q., Wang W., Xiang H. (2018). New insight into the mechanism underlying the silk gland biological process by knocking out fibroin heavy chain in the silkworm. BMC Genom..

[122] Zhang X., Xia L., Day B.A., Harris T.I., Oliveira P., Knittel C., Licon A.L., Gong C., Dion G., Lewis R.V. (2019). CRISPR/Cas9 Initiated Transgenic Silkworms as a Natural Spinner of Spider Silk. Biomacromolecules.

[123] Michlewski G., Cáceres J.F. (2019). Post-transcriptional control of miRNA biogenesis. RNA.

[124] Ling L., Ge X., Li Z., Zeng B., Xu J., Chen X., Shang P., James A.A., Huang Y., Tan A. (2015). MiR-2 family targets awd and fng to regulate wing morphogenesis in *Bombyx mori*. RNA Biol..

[125] Liu Z., Xu J., Ling L., Luo X., Yang D., Yang X., Zhang X., Huang Y. (2020). miR-34 regulates larval growth and wing morphogenesis by directly modulating ecdysone signalling and cuticle protein in *Bombyx mori*. RNA Biol..

[126] Mahlapuu M., Håkansson J., Ringstad L., Björn C. (2016). Antimicrobial Peptides: An Emerging Category of Therapeutic Agents. Front. Cell. Infect. Microbiol..

[127] Furukawa S., Tanaka H., Ishibashi J., Imanishi S., Yamakawa M. (2009). Functional Characterization of a Cactus Homolog from the Silkworm *Bombyx mori*. Biosci. Biotechnol. Biochem..

[128] Park J.W., Yu J.H., Kim S.W., Kweon H.Y., Choi K.H., Kim S.R. (2018). Enhancement of antimicrobial peptide genes expression in Cactus mutated *Bombyx mori* cells by CRISPR/Cas9. Int. J. Ind. Entomol..

[129] Gan B.H., Gaynord J., Rowe S.M., Deingruber T., Spring D.R. (2021). The multifaceted nature of antimicrobial peptides: Current synthetic chemistry approaches and future directions. Chem. Soc. Rev..

[130] Yang H., Wang M., Zhang P., Sabhat A., Malik F.A., Bhaskar R., Zhou F., Li X.H., Hu J.-B., Sun C.G. (2011). Cloning and characterization of the *Bombyx mori* ecdysone oxidase. Arch. Insect Biochem. Physiol..

[131] Li Z., You L., Zeng B., Ling L., Xu J., Chen X., Zhang Z., Palli S.R., Huang Y., Tan A. (2015). Ectopic expression of ecdysone oxidase impairs tissue degeneration in *Bombyx mori*. Proc. R. Soc. B Biol. Sci..

[132] Yamamoto K., Nagaoka S. (2017). Identification and localization of a novel ecdysone oxidase in the silkworm, *Bombyx mori*. J. Insect Biotechnol. Sericol..

[133] Tettamanti G., Casartelli M. (2019). Cell death during complete metamorphosis. Philos. Trans. R. Soc. B Biol. Sci..

[134] Zhang Z., Liu X., Shiotsuki T., Wang Z., Xu X., Huang Y., Li M., Li K., Tan A. (2017). Depletion of juvenile hormone esterase extends larval growth in *Bombyx mori*. Insect Biochem. Mol. Biol..

[135] Liu Y., Ma S., Chang J., Zhang T., Chen X., Liang Y., Xia Q. (2019). Programmable targeted epigenetic editing using CRISPR system in *Bombyx mori*. Insect Biochem. Mol. Biol..

[136] Xing W., Ma S., Liu Y., Xia Q. (2019). CRISPR/dCas9-mediated imaging of endogenous genomic loci in living *Bombyx mori* cells. Insect Sci..

[137] Brady D., Saviane A., Cappellozza S., Sandrelli F. (2020). An Efficient Workflow for Screening and Stabilizing CRISPR/Cas9-Mediated Mutant Lines in *Bombyx mori*. Methods Protoc..

[138] Nartey M.A., Sun X., Qin S., Hou C.X., Li M.W. (2020). CRISPR/Cas9-based knockout reveals that the clock gene timeless is indispensable for regulating circadian behavioral rhythms in *Bombyx mori*. Insect Sci..

[139] Yu Y., Liu X., Ma X., Zhang Z., Wang T., Sun F., Hou C., Li M. (2020). A palmitoyltransferase Approximated gene Bm-app regulates wing development in *Bombyx mori*. Insect Sci..

[140] Zeng B., Huang Y., Xu J., Shiotsuki T., Bai H., Palli S.R., Huang Y., Tan X.A. (2017). The FOXO transcription factor controls insect growth and development by regulating juvenile hormone degradation in the silkworm, *Bombyx mori*. J. Biol. Chem..

[141] Hong J.W., Jeong C.Y., Yu J.H., Kim S.B., Kang S.K., Kim S.W., Kim N.S., Kim K.Y., Park J.W. (2020). *Bombyx mori* kynurenine 3-monooxygenase gene editing and insect molecular breeding using the clustered regularly interspaced short palindromic repeat/CRISPR associated protein 9 system. Biotechnol. Prog..

[142] Xu X., Zhang Z., Yang Y., Huang S., Li K., He L., Zhou X. (2018). Genome editing reveals the function of Yorkie during the embryonic and early larval development in silkworm, *Bombyx mori*. Insect Mol. Biol..

[143] Zhang Z.J., Liu X.J., Yu Y., Yang F.Y., Li K. (2020). The receptor tyrosine kinase torso regulates ecdysone homeostasis to control developmental timing in *Bombyx mori*. Insect Sci..

[144] Xu X., Wang Y., Liu Z., Wang Y., He L., Li K., Huang Y. (2021). Disruption of egg-specific protein causes female sterility in *Bombyx mori*. Insect Sci..

[145] Zhu L., Mon H., Xu J., Lee J.M., Kusakabe T. (2015). CRISPR/Cas9-mediated knockout of factors in non-homologous end joining pathway enhances gene targeting in silkworm cells. Sci. Rep..

[146] Gao Y., Liu Y.C., Jia S.Z., Liang Y.T., Tang Y., Xu Y.S., Kawasaki H., Wang H.B. (2020). Imaginal disc growth factor maintains cuticle structure and controls melanization in the spot pattern formation of *Bombyx mori*. PLoS Genet..

[147] Sun L., Zhang Z., Zhang R., Yu Y., Yang F., Tan A. (2020). Molecular Disruption of Ion Transport Peptide Receptor Results in Impaired Water Homeostasis and Developmental Defects in *Bombyx mori*. Front. Physiol..

[148] Liu Z.L., Xu J., Ling L., Zhang R., Shang P., Huang Y.P. (2018). CRISPR disruption of TCTP gene impaired normal development in the silkworm *Bombyx mori*. Insect Sci..

[149] Zhang Z., Zhang S.S., Niu B.L., Ji D.F., Liu X.J., Li M.W., Bai H., Palli S.R., Wang C.Z., Tan A.J. (2019). A determining factor for insect feeding preference in the silkworm, *Bombyx mori*. PLoS Biol..

[150] Bi H., Xu X., Li X., Zhang Y., Huang Y., Li K., Xu J. (2019). CRISPR Disruption of BmOvo Resulted in the Failure of Emergence and Affected the Wing and Gonad Development in the Silkworm *Bombyx mori*. Insects.

[151] Fujii T., Banno Y. (2018). Enlargement of egg size by CRISPR/Cas9-mediated knockout of a sex-linked gene in the silkworm, *Bombyx mori*. J. Insect Biotechnol. Sericol..

[152] Zhang Z., Aslam A.F., Liu X., Li M., Huang Y., Tan A. (2015). Functional analysis of Bombyx Wnt1 during embryogenesis using the CRISPR/Cas9 system. J. Insect Physiol..

[153] Li K., Tian L., Guo Z., Guo S., Zhang J., Gu S., Palli S.R., Cao Y., Li S. (2016). 20-Hydroxyecdysone (20E) Primary Response Gene E75 Isoforms Mediate Steroidogenesis Autoregulation and Regulate Developmental Timing in *Bombyx*. J. Biol. Chem..

[154] Liu Q., Liu W., Zeng B., Wang G., Hao D., Huang Y. (2017). Deletion of the *Bombyx mori* odorant receptor co-receptor (*BmOrco*) impairs olfactory sensitivity in silkworms. Insect Biochem. Mol. Biol..

[155] Baddeley H.J.E., Isalan M. (2021). The Application of CRISPR/Cas Systems for Antiviral Therapy. Front. Genome Ed..

[156] Murphy B.G., Wolf T., Vogel H., Castillo D., Woolard K. (2020). An RNA-Directed Gene Editing Strategy for Attenuating the Infectious Potential of Feline Immunodeficiency Virus-Infected Cells: A Proof of Concept. Viruses.

[157] Fareh M., Zhao W., Hu W., Casan J.M., Kumar A., Symons J., Zerbato J.M., Fong D., Voskoboinik I., Ekert P.G. (2021). Reprogrammed CRISPR-Cas13b suppresses SARS-CoV-2 replication and circumvents its mutational escape through mismatch tolerance. Nat. Commun..

[158] Yao Q., Li M.W., Wang Y., Wang W.B., Lu J., Dong Y., Chen K.P. (2003). Screening of molecular markers for NPV resistance in *Bombyx mori* L. (Lep., Bombycidae). J. Appl. Entomol..

[159] Guo H., Zhang B., Zheng X., Sun J., Guo H., Li G., Zhao G., Xu A., Qian H. (2021). Pathogenicity Detection and Genome Analysis of Two Different Geographic Strains of BmNPV. Insects.

[160] Isobe R., Kojima K., Matsuyama T., Quan G.X., Kanda T., Tamura T., Sahara K., Asano S.I., Bando H. (2004). Use of RNAi technology to confer enhanced resistance to BmNPV on transgenic silkworms. Arch. Virol..

[161] Qiong Y., Dong Xu X., Qing Rong L., Yang X., Ming Qiang Y. (2017). Standard method for detecting *Bombyx mori* nucleopolyhedrovirus disease-resistant silkworm varieties. Rev. Bras. Entomol..

[162] Chen S., Hou C., Bi H., Wang Y., Xu J., Li M., James A.A., Huang Y., Tan A. (2017). Transgenic Clustered Regularly Interspaced Short Palindromic Repeat/Cas9-Mediated Viral Gene Targeting for Antiviral Therapy of *Bombyx mori* Nucleopolyhedrovirus. J. Virol..

[163] Dong Z., Hu Z., Qin Q., Dong F., Huang L., Long J., Chen P., Lu C., Pan M. (2018). CRISPR/Cas9 Mediated Disruption of the immediate early-0 and 2 as a Therapeutic Approach to *Bombyx mori* Nucleopolyhedrovirus in Transgenic Silkworm. Insect Mol. Biol..

[164] Dong Z., Qin Q., Hu Z., Chen P., Huang L., Zhang X., Tian T., Lu C., Pan M. (2019). Construction of a One-Vector Multiplex CRISPR/Cas9 Editing System to Inhibit Nucleopolyhedrovirus Replication in Silkworms. Virol. Sin..

[165] Xu X., Bi H., Wang Y., Li X., Xu J., Liu Z., He L., Li K., Huang Y. (2019). Disruption of the ovarian serine protease (Osp) gene causes female sterility in *Bombyx mori* and Spodoptera litura. Pest Manag. Sci..

[166] Sledzinski P., Nowaczyk M., Olejniczak M. (2020). Computatonal Tools and Resources Supporting CRISPR-Cas Experiments. Cells.

